# Detection of Aflatoxin B1 in Wheat Based on Nucleic Aptamer Chemiluminescence Sensor

**DOI:** 10.3390/s25040988

**Published:** 2025-02-07

**Authors:** Zebing Zhang, Caizhang Wu, Zhike Zhao

**Affiliations:** College of Electrical Engineering, Henan University of Technology, Zhengzhou 450001, China; zhangzebing@stu.haut.edu.cn (Z.Z.); wucaizhang@haut.edu.cn (C.W.)

**Keywords:** aflatoxin B_1_ detection, aptamer, chemiluminescence sensor, optical fiber, wheat

## Abstract

In this study, we developed a low-cost, high-sensitivity chemiluminescence competitive aptamer sensor for the detection of aflatoxin B_1_ (AFB_1_) in wheat samples. The optical fiber sensor was self-made, and it utilized biotin and streptavidin (SA) link aptamer and horseradish peroxidase (HRP) for the chemiluminescence detection, achieving competitive assay between the AFB_1_ and AFB_1_ antigen. We adjusted the experimental conditions of the sensor base on the date of optimization of the experimental conditions and chose coated antigens on the surface of carboxyl magnetic particles. Under conditions optimized by testing key parameters, the assay results showed that the chemiluminescence intensity and AFB_1_ concentration demonstrated a strong linear relationship (R^2^ = 0.995), the dynamic range was from 0.1 to 10 ng/mL with a detection limit of 0.09 ng/mL, and the aptamer exhibited good specificity and anti-interference ability. Testing the wheat samples showed that the spiked recovery rate ranged from 79.19% to 113.21%. The sensor possesses characteristics of low detection limits, simple manufacturing methods, and affordability, providing a novel solution for the development of low-cost and high-sensitivity AFB_1_ detection equipment.

## 1. Introduction

Food security is directly related to people’s quality of life and health level, so it has attracted public attention. Aflatoxin is one of the major threats to food security in the whole process of food storage. Aflatoxin has a strong pollution ability, which is easy to cause harm to grain, and this phenomenon is more serious in hot and humid areas [1,2]. The basic structure of aflatoxin is dihydrocoumarin, which is composed of difuran ring and *oxynaphthalene ortho ketone*. There are many kinds of aflatoxins, of which B, G, and M series are the most prominent ones, namely AFB_1_, AFB_2_, AFG_1_, AFG_2_, AFM_1_, and AFM_2_ [3]. Among them, AFB_1_ is mainly a secondary metabolite produced by *Aspergillus flavus* and *Aspergillus parasiticus* [4,5]. Aflatoxin B_1_ is widely found in peanut, corn, soybean, wheat, and other food and agricultural products [6]. When food is contaminated by AFB_1_, it can enter human and animal bodies through the food chain, inhibit the synthesis of proteins; enzymes, organic substances, cellular DNA, and RNA in human and animal bodies; and then interfere with various metabolic processes, causing serious harm to human and animal health [7,8], clinical investigations have found that AFB_1_ can be transformed into AFM_1_, AFBO, and other harmful substances in the human body, which can cause genetic and protein expression disorders, lead to metabolic disorders, and even lead to cell distortion or cancer, causing serious damage to the human body [9]. In 1993, the Cancer Research Institute of the World Health Organization (WHO) classified it as a class I carcinogen (for humans). The European Commission strictly stipulated that the permitted level of aflatoxin B_1_ be set at ≤2 μg/kg in some cereals and their products 2 μg/kg; the maximum limit of aflatoxin in China’s current standard GB 2761-2017 is 5–20 μg/kg [10,11,12]. To ensure the safety of grain storage and people’s health and effectively monitor aflatoxin pollution, there is an urgent need for an AFB_1_ toxin detection system applied to the grain industry. At present, a variety of detection methods have been reported. Common methods are mainly large-scale instrument detection, including high-performance liquid chromatography (HPLC) [13], fluorescence (FL) [14], electrochemiluminescence (ECL) [15], photo electrochemistry (PEC) [16], surface enhanced Raman spectroscopy (SERS) [17], optical fibers etc. [18].

In recent years, an innovative based method has been developed in the field of toxin detection. Aptamer is a single-stranded oligonucleotide sequence, which is screened in vitro through the systematic evolution of ligands by exponential enrichment (SELEX), and it has the advantages of low cost, high stability, and strong specificity [19]. Compared with antibodies, aptamers have stronger tolerance to environmental changes, better stability, and advantages such as fast chemical synthesis and low cost, so aptamers are also called the “artificial antibody” [20,21,22]. Aptamers have a simple structure and are easy to modify. Various substances required for experiments can be added at the 5′ end or 3′ end. For example, Li et al. [23] designed an experimental method for the combination of aptamer and SA-HRP based on the aptamer modified with biotin and established a simple and sensitive DNA magnetic particle chemiluminescence method for the detection of avian influenza H_1_N_1_ virus DNA. Shim et al. [24] developed a chemiluminescence competitive aptamer assay for AFB_1_ using a hemin/G-quadruplex HRP-DNAzyme linked with an aptamer specific to AFB_1_. Yao et al. [25] developed a chemiluminescence aptamer sensor based on the HRP-catalyzed chemiluminescence reaction of luminol. They used a mixed chain reaction (HCR) signal amplification strategy to improve detection sensitivity and employed magnetic separation techniques to further reduce background signals. In 2022, Hu et al. and others used multimode optical fiber combined with an optical fiber photon counting probe to detect the target signal and designed an aflatoxin B_1_ optical fiber detection system based on photoelectric detection technology [26].

The conventional detection method coats the antigen on the microplate, the separation performance of the microplate is weak, and the steps are cumbersome, while the large surface area to volume ratio of the magnetic particles increases the enrichment of the material on its surface and improves the separation efficiency. Modification of carboxyl, amino, thiol, and other active groups on the surface of magnetic particles can provide a wide choice for the enrichment of different target substances. Therefore, new research began to use magnetic particles combined with aptamers, use the superparamagnetism of magnetic particles, use magnetic particles to separate and purify target substances from various complex substances, strengthen the separation performance of the experiment, and omit centrifugation and other operations. In 2023, Lu et al. [27] developed a method for detecting aflatoxin B_1_ (AFB_1_) in plant protein meat by SERS aptamer sensor composed of magnetic nanoparticles of ferro-tetroxide.

To reduce the cost of sensor development and improve the sensitivity of the sensor, we designed a novel aptamer chemiluminescence sensor based on the aptamer. In this work, we investigated the uses of biotin, SA-HRP, and Luminol. And we examined the optimization of experimental conditions, specificity, and anti-interference ability; assessed the efficiency of the developed assay included its use for AFB_1_ detection in wheat samples, and compared the sensors with similar methods.

## 2. Materials and Methods

### 2.1. Materials and Reagents

AFB_1_-related mycotoxins (AFB_2_, AFG_1_, and ochratoxin A (OTA)), bovine serum albumin (BSA), Luminol, streptavidin conjugated with horseradish peroxidase (SA-HRP), P-iodophenol (PIP), TE buffer, 1× solution (low EDTA), phosphate buffer (PBS) (pH 7.4), methanol, H_2_O_2_, sodium N-hydroxy succinimide (NHS), 1-(3-dimethylaminopropyl)-3-ethylcarbodiimide (EDC), and Tween 20 were purchased from Sangon Biotech. Co., Ltd. (Shanghai, China). Ultrapure water for experiment was purchased from Ruixi chemical treatment plant (Jiaxing, China); aflatoxin B_1_ antigen (AFB_1_-OVA) was purchased from Anti Biological Technology Co., Ltd. (Shenzhen, China); carboxy magnetic particles (2 μm) were purchased from Suzhou Beaver Biosciences Inc. (Suzhou, China); wheat samples were purchased from local farmers’ market (Zhengzhou, China). The DNA sequences were synthesized by Sangon Biotech. Co., Ltd.; (Shanghai, China) and purified using high-performance liquid chromatography (HPLC). The DNA sequences are as follows:

GTTGGGCACGTGTTGTCTCTCTGTGTCTCGTGCCCTTCGCTAGGCCCACA; from the 5′ end to the 3′ end, the 5′ end is modified with biotin.

### 2.2. Preparation of AFB_1_ Standard Solution and AFB_1_-Spiked Wheat Samples

We prepared AFB_1_ standard solution at different concentrations (0, 0.1, 0.3, 1, 3, and 10 ng/mL) in methanol/water (10:90, *v*/*v*). The cross-reactivity of AFB_1_-related compounds (AFB_2_, AFG_1_, and OTA) was also determined, with each compound prepared at a concentration of 10 ng/mL in methanol/water (10:90, *v*/*v*).

First, 20.0 g of wheat sample was weighted and crushed into powder, and 1.0 g of wheat flour sample was placed in a 50 mL centrifuge tube. Next, 25 mL of methanol/water (70:30, *v*/*v*) was added into the centrifuge tube for 20 min oscillation extraction and centrifuged for 5 min at 12,000 R/min. Then, 30 μL of supernatant were dried and mixed with 100 μL of PBS (pH 7.4), soaked for 3 min, and mixed for 20 times to obtain the sample detection solution; the obtained sample solutions (0, 0.5, 1, 2, 5, and 10 μg/kg) were stored at 4 °C for future use [28,29].

### 2.3. AFB_1_-OVA Coated

Next, 50 μL of the carboxyl magnetic particle solution with a concentration of 10 mg/mL was placed in a centrifuge tube, vortexed, and mixed for 10 s. It was placed on a magnetic separation rack for magnetic separation for 3 min, and the supernatant was removed. Then, 100 μL of EDC (10 mg/mL) and 100 μL of NHS (10 mg/mL) were added to the new configuration and activated at 25 °C for 20 min, and 25 ng of AFB_1_-OVA antigen in 100 μL PBS were added to the magnetic particle solution and incubated at 37 °C for 1 h. After washing three times with PBST (pH 7.4, containing 0.1% Tween 20), the magnetic particle solution was blocked with 200 μL 1% BSA and kept overnight at 4 °C, and then, it was washed four times with PBST (pH 7.4, containing 0.1% Tween 20).

### 2.4. Chemiluminescence Competitive Aptamer Assay

We prepared an aptamer solution in TE buffer solution for later use. Then, 100 μL of aflatoxin B_1_ (AFB_1_) standard solution or 100 μL of AFB_1_ sample solution and 100 μL of aptamer solution (0.6 nM) were added to a centrifuge tube and incubated at 30 °C for 15 min for competitive steps. After completion, we placed the magnetic particle solution on a magnetic separation rack for magnetic separation for 3 min and washed 10 times with PBST (pH 7.4, containing 0.1% Tween 20).

After the binding step between the aptamer and aflatoxin AFB_1_ was completed, 100 μL of PBST (pH 7.4, containing 0.1% Tween 20) mixed solution containing 200 ng/mL SA-HRP was added to each centrifuge tube, and the reaction was gently shaken at 37 °C for 30 min. After the reaction was complete, we placed the magnetic particle solution on a magnetic separation rack for magnetic separation for 3 min, washed it 5 times with PBST (pH 7.4, containing 0.1%, Tween 20), and washed it once with PBS (pH 7.4). The mixed solution of magnetic particle aptamers obtained after completing the binding reaction was transferred to a CL reaction tube and the washing buffer removed.

Then, 200 μL of CL reaction solution (2 mM luminol, 0.5 mM P-iodophenol (PIP), and 2 mM H_2_O_2_) was added to a centrifuge tube in a PBS (pH 7.4) environment. Finally, optical fibers were used to detect solutions in centrifuge tube, and the CL intensity was measured using a sensor.

## 3. Results

### 3.1. Principle of Sensor for AFB_1_ Detection

**Figure 1:**
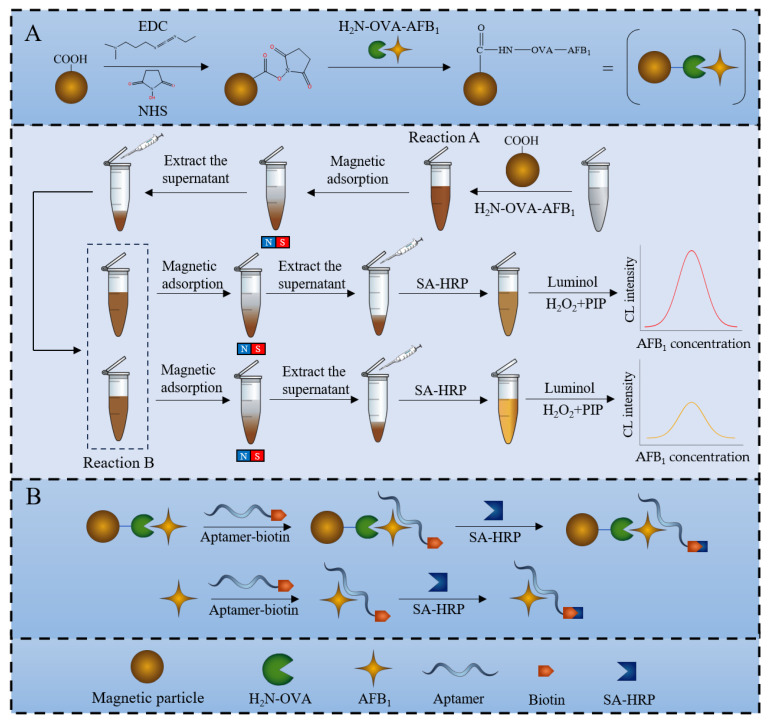
Schematic illustration of AFB_1_ detection using the chemiluminescence sensor. (**A**) The process of coating antigen on magnetic particles. (**B**) Competitive binding aptamer mechanism between AFB_1_ and AFB_1_-OVA.

A detailed depiction of AFB_1_ detection is presented in Figure 1: (A) Firstly, NHS and EDC reagent are added to the magnetic particle solution to activate the magnetic particles. After that, AFB_1_-OVA is coated on the magnetic particles. (B) Following this, the aptamer and AFB_1_ are added to the solution and a competitive step between AFB_1_ and AFB_1_-OVA will start. After the competition is completed, part of the aptamer will bind to AFB_1_-OVA and form the OVA-AFB1–aptamer conjugate, and this part will be immobilized on the magnetic particle. Another part of the aptamer will bind to the free AFB_1_ and form the OVA-AFB1–aptamer conjugate. These two components, the OVA-AFB_1_–aptamer conjugate and AFB_1_–aptamer conjugate, are separated by magnetic force, and then, the AFB_1_–aptamer conjugate in the supernatant is removed. Afterwards, SA-HRP is added to the solution, and SA-HRP binds to the aptamer through biotin on the aptamer, forming the OVA-AFB_1_–aptamer–SA-HRP conjugate. Lastly, we add the CL reaction solution (luminol, P-iodophenol (PIP), and H_2_O_2_). The CL solution will release energy in the form of blue shimmer radiation [30] and also will detect chemiluminescence intensity with the sensor.

The experiment is based on indirect competition law, and the concentration of AFB_1_ was determined by detecting the number of magnetic particle–antigen–aptamer complexes. When the concentration of AFB_1_ is high, the aptamer will be more inclined to bind to the free AFB_1_ and be cleared out of the test tube with the supernatant. On the contrary, the aptamer will be more inclined to bind with AFB_1_ antigen and immobilize it together with the antigen on magnetic particles. When SA-HRP is added, and the AFB_1_ concentration is high, more aptamers will be cleared out of the test tube with AFB_1_, reducing the chance of SA-HRP binding to aptamers. The SA-HRP fixed on magnetic particles will decrease, and the chemiluminescence intensity will also weaken. On the contrary, when the concentration of AFB_1_ is low, the SA-HRP fixed on the magnetic particles will increase, and the detected chemiluminescence intensity will also increase. In theory, the chemiluminescence intensity is inversely proportional to the concentration of AFB_1_.

### 3.2. Sensor Workflow

**Figure 2:**
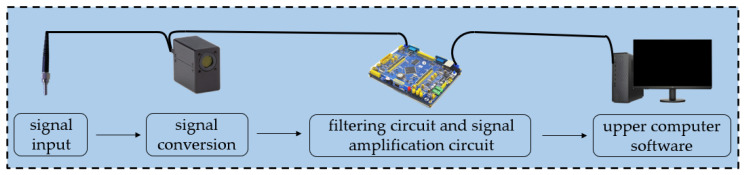
Sensor workflow diagram.

This design uses a self-made fiber optic sensor to detect the chemiluminescence intensity of the AFB_1_ solution at different concentrations. The sensor system includes an optical signal input module, signal conversion module, filtering circuit, signal amplification circuit, and upper computer software in the computer. The circuit workflow diagram is shown in Figure 2.

The optical signal input module, also known as optical fiber, transmits the detected chemiluminescence signal to the signal input module. The signal input module includes a photon counter and a microcontroller. The photon counter converts the number of detected photons into an equal number of standard TTL (Transistor–Transistor Logic) signals and outputs them to the microcontroller. The microcontroller counts the standard TTL signals and outputs the number of photons, which is the chemiluminescence intensity generated in the experiment. After passing through the circuit and signal amplification circuit, the data are finally transmitted to the computer.

To avoid errors caused by natural light, the experiment needs to be conducted in a dark room. And due to the lack of transparency of magnetic particles themselves, the detected chemiluminescence intensity may be slightly weakened compared to conventional methods, which needs to be considered when processing data. Moreover, the uneven concentration of magnetic particles at different positions in the solution can easily cause errors during detection, and it is necessary to control the errors by controlling the concentration of magnetic particles by centrifuging and mixing. The results detected by the light intensity detection equipment show that before the experiment, the number of photons in the darkroom was within 100. When the AFB_1_ concentration was 0 ng/mL, the number of photons remained stable at around 40,000. When the AFB_1_ concentration was 10 ng/mL, the number of photons remained stable at around 4500. Based on these data, errors can be minimized to the greatest extent possible.

### 3.3. Assessment of Detection Performance for Different Coating Modes

**Figure 3:**
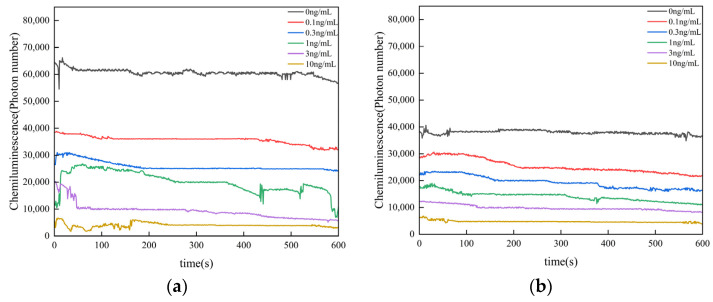
AFB_1_ detection curve (**a**) with coated antigens on microplate and (**b**) AFB_1_ detection curve with coated antigens on magnetic particles.

To verify the detection accuracy of the sensor for AFB_1_ at concentrations of 0, 0.1, 0.3, 1, 3, and 10 ng/mL under different conditions of magnetic particles and microplates, this study conducted two sets of experiments with coated antigens on microplates and magnetic particles, respectively. The sensor detection curve shows that coating the antigen on a microplate resulted in higher chemiluminescence intensity, but the linear curve between chemiluminescence intensity and toxin concentration fluctuated more and had lower accuracy compared to the latter. Coating the antigen onto magnetic particles resulted in a certain degree of decrease in chemiluminescence intensity, but the linear curve showed better stability and accuracy. The specific data are shown in Figure 3.

### 3.4. Optimization of Experimental Conditions

**Figure 4:**
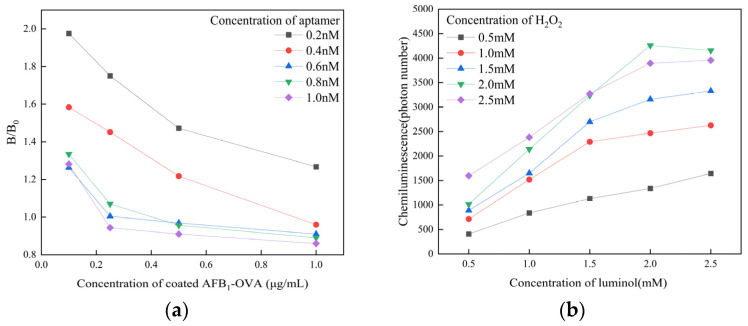
(**a**) The selection of concentration of AFB_1_-OVA and aptamer and (**b**) the selection of concentration of H_2_O_2_ and luminol.

The concentration of the aptamer, concentration of the coated antigen, and pH value will affect the sensitivity of the detection system. To select the optimal concentration of aptamer and coated antigen, we conducted a checkboard titration experiment. The data are shown in Figure 4a. The experiment was conducted with concentrations of AFB_1_-OVA of 0.1 μg/mL, 0.25 μg/mL, 0.5 μg/mL, and 1 μg/mL, and the corresponding concentrations of aptamer were 0.2, 0.4, 0.6, 0.8, and 1 nM, respectively. The AFB_1_ concentration of control sample *B*_0_ was 10 ng/mL, and the sensitivity of the sensor was calculated by *B*/*B*_0_. The test result of *B*/*B*_0_ was 1.005, so the sensor obtained the most accurate experimental data when the aptamer concentration was 0.6 nM and the coated AFB_1_-OVA concentration was 0.25 μg/mL. Consequently, 0.6 nM was determined as the optimal aptamer concentration, and 0.25 μg/mL was determined as the optimal concentration of coated AFB_1_-OVA.

The optimal concentrations of luminol and H_2_O_2_ were determined when the concentration of magnetic particles was 10 μg/mL; the data are shown in Figure 4b. The concentrations of H_2_O_2_ were 0.5, 1.0, 1.5, 2, and 2.5 nM, and the corresponding concentrations of luminol were 0.5, 1.0, 1.5, 2, and 2.5 nM, respectively. The data in Figure 4b show that the highest chemiluminescence intensity was obtained by the sensor at both luminal and hydrogen peroxide concentrations of 2 nM.

**Figure 5:**
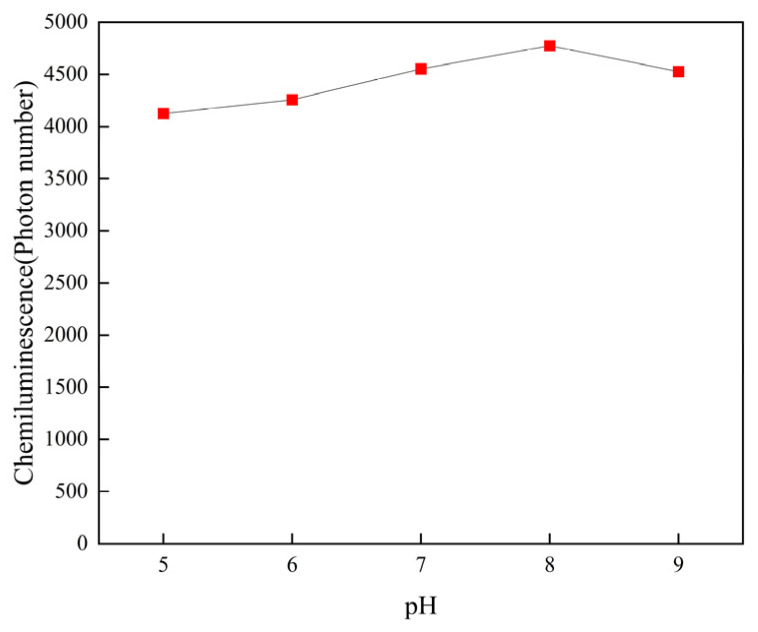
Effects of pH.

The pH value of the aptamer solution is a key parameter, potentially resulting in the instability of the sensor, so we utilized different pH environments in the step of ligand binding to AFB_1_, as shown in Figure 5. As the pH value (pH = 5, 6, 7, 8, and 9) of the aptamer solution increased, there was no significant change in the chemiluminescence intensity. This proves the acid and alkali resistance of the aptamer. Due to the relatively highest chemiluminescence intensity at pH value = 7, we ultimately chose to adjust the pH value to 7.0.

### 3.5. Detection of AFB_1_ by the Aptamer Sensor

**Figure 6:**
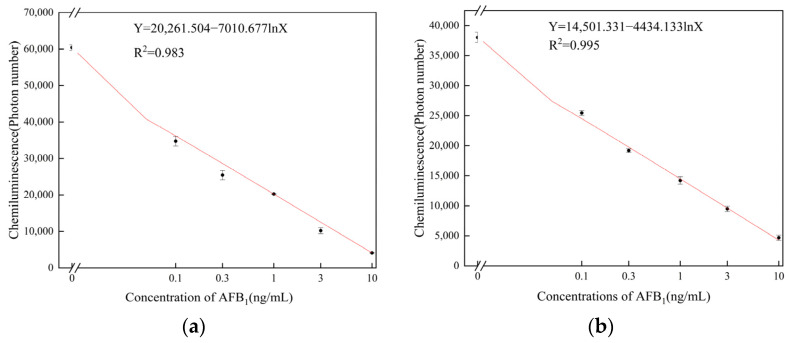
Sensor fitting curve diagram at different concentrations of AFB_1_ (**a**) with coated antigen on microplate and (**b**) with coated antigen on magnetic particles.

To verify the performance of the sensor under the two different coating conditions of microplate and magnetic particle, we used sensor tests in both situations. The chemiluminescence intensity of the antigen coated on microplate is shown in Figure 6a, and the chemiluminescence intensity of the antigen coated on magnetic particles is shown in Figure 6b. The chemiluminescence intensity under both coating conditions exhibited a notable decrease with an increasing AFB_1_ concentration within the range of 0.1–10 ng/mL. The dynamic curves obtained under the condition with coated antigen on magnetic particles showed higher consistency than those obtained with coated antigen on microplate. And coating the antigen on magnetic particles resulted in a higher R-squared value; the curve equation for coated antigen on microplate is as follows:

$$y=20,261.5-7010.7ln\;x,\;R^{2}=0.983,$$

The curve equation for coated antigen on magnetic particles is as follows:

$$y=14,501.3-4434.1ln\;x,\;R^{2}=0.995.$$

The above results demonstrate that coating antigens on magnetic particles shows a better detection performance.

### 3.6. Sensor Specificity and Anti-Interference

**Figure 7:**
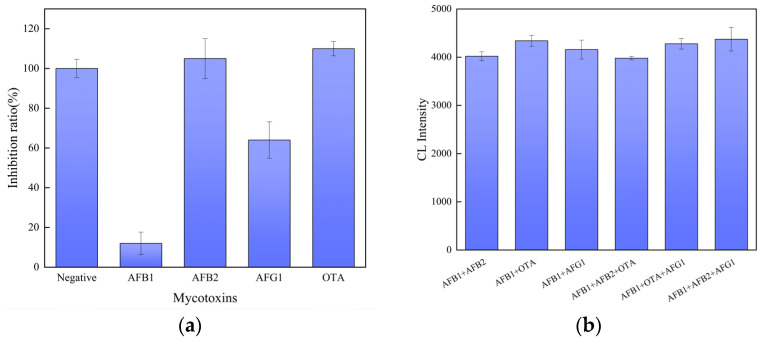
Inhibition ratio (**a**) and the chemiluminescence intensity of the sensor to AFB_1_, AFB_2_, AFB_1_+ AFB_2_, AFG_1_, and AFB_1_+ AFG_1_ (**b**) (n = 3).

In order to investigate the specificity and anti-interference of the sensor, under the same conditions, a methanol/water (10:90, *v*/*v*) solution without AFB_1_ content was used as the negative sample in the specificity and anti-interference ability tests of the sensor, and several common fungal toxins were introduced: AFB_2_, AFG_1_, and OTA were used as positive samples, and 100 μL of the above fungal toxin solutions with a concentration of 10 ng/mL were taken for detection. We assumed that the number of photons detected from the negative sample was $X_{F}$, and the number of photons detected from the positive sample was $X_{P}$; the inhibition ratio was $\;I=X_{P}/X_{F}$; the sensor specificity detection results are shown in Figure 7a. The sensor had the lowest inhibition ratio for AFB_1_, and there was no significant response to other fungal toxins; this proves that the sensor has good specificity for AFB_1_.

The anti-interference ability of the prepared sensor was also crucial for assessing its performance. After mixing the AFB_1_ solution with equal amounts of the solution of other fungal toxins, we used sensors to detect the mixed solutions. The detected chemiluminescence intensity of the mixed solution showed the anti-interference ability of the sensor. The anti-interference detection results are shown in Figure 7b, and the chemiluminescence intensity of the sensor to different mixed solutions was basically similar, which proves that the sensor has good anti-interference ability.

### 3.7. Application of Sensor in Wheat Samples

To further validate the application and feasibility of the sensor for AFB_1_ detection, we prepared the spiked wheat sample using a previously established method [27,28]. According to Table 1, using spiked wheat samples with AFB_1_ concentrations of 0, 0.5, 1, 2, 5, and 10 μg/kg as the negative sample, the spiked recovery rate of AFB_1_ in the wheat sample solution was 79.19% to 113.21%, indicating that the aptamer sensor could be employed for AFB_1_ detection in wheat samples.

### 3.8. Comparison of the Chemiluminescence Aptamer Sensor

Before this study, there were many sensors using aptamer or chemiluminescence methods to detect AFB_1_, and several relevant studies about chemiluminescence assays or aptamer assays are listed in Table 2. Compared to the method using chemiluminescence for AFB_1_ detection, this study yielded a lower detection limit and similar detection range. Compared to our study, sensors using electrochemical and fluorescence methods have a wider detection range, but their detection limits and ranges are lower than those of our design. And compared with other methods in Table 2, the material price of this design is lower, and the manufacturing method is simpler.

## 4. Conclusions

In this study, we developed a chemiluminescence indirect competitive aptamer sensor for aflatoxin B_1_ (AFB_1_) utilizing aptamer-linked streptavidin–horseradish peroxidase (SA-HRP), and we applied the sensor to wheat samples. The sensor is capable of specifically recognizing AFB_1_ and accurately measuring its concentration. It operates on the principle that AFB_1_ and AFB_1_ antigens compete for binding to the aptamer, with the chemiluminescence intensity detected through a custom-built chemiluminescence detection device. The high specificity and binding efficiency of the aptamer for AFB_1_ were confirmed through both simulations and experimental validations. The results indicated that the change in chemiluminescence intensity is linear within the concentration range of 0.1–10 ng/mL. For wheat samples spiked with AFB_1_ at a concentration of 10 ng/mL, the recovery rate after three repeated detections ranged from 79.19% to 113.21%. Compared to other sensors, this design features a low cost and a straightforward manufacturing process. Additionally, the experimental results demonstrate that this sensor exhibits high accuracy and a low detection limit. This study holds innovative significance for the advancement of low-cost and high-sensitivity AFB_1_ detection equipment.

## Figures and Tables

**Table 1:** Determination of recovery of AFB_1_ in wheat samples (n = 3).

Added AFB_1_/(μg/kg)	Found AFB_1_/(μg/kg)	Recovery/%
0	<LOD	-
0.5	0.396 ± 0.050	79.19 ± 10.01
1	0.985 ± 0.127	98.53 ± 12.69
2	2.264 ± 0.106	113.21 ± 5.31
5	4.628 ± 0.351	92.56 ± 7.02
10	10.929 ± 0.429	109.29 ± 4.29

**Table 2:** Comparison of the related chemiluminescence aptamer sensor.

Methods		LOD	Linear (L)/Dynamic (D) Range	Tested Samples	Reference
Chemiluminescence	Aptamer	0.09 ng/mL	(D) 0.1–10 ng/mL	Wheat	This work
Chemiluminescence	Aptamer	0.20 ng/mL	(L) 0.5–40 ng/mL	Peanut and milk	[25]
Chemiluminescence	Antibody	0.53 ng/mL	(D) 0.1–10 ng/mL	Wheat	[31]
Fluorescent	Aptamer	1.6 ng/mL	(L) 5–100 ng/mL	Rice cereal	[32]
Fluorescent	Aptamer	0.35 ng/mL	(L) 0–180 ng/mL	Corn, milk, and rice	[33]
Fluorescent	Aptamer	0.62 ng/mL	(D) 0.62–312.27 ng/mL	Serum, urine, wine, and beer	[34]
Electrochemiluminescence	Aptamer	0.17 ng/mL	(L) 0.50–200.00 ng/mL	Corn	[35]

## Data Availability

The data presented in this study are available on request from the corresponding authors.
